# Predicting plaque vulnerability change using intravascular ultrasound + optical coherence tomography image-based fluid–structure interaction models and machine learning methods with patient follow-up data: a feasibility study

**DOI:** 10.1186/s12938-021-00868-6

**Published:** 2021-04-06

**Authors:** Xiaoya Guo, Akiko Maehara, Mitsuaki Matsumura, Liang Wang, Jie Zheng, Habib Samady, Gary S. Mintz, Don P. Giddens, Dalin Tang

**Affiliations:** 1 School of Science, Nanjing University of Posts and Telecommunications, Nanjing, 210023 China; 2 Department of Mathematics, Southeast University, Nanjing, 210096 China; 3 The Cardiovascular Research Foundation, Columbia University, New York, NY 10022 USA; 4 School of Biological Science and Medical Engineering, Southeast University, Nanjing, 210096 China; 5 Mallinckrodt Institute of Radiology, Washington University, St. Louis, MO 63110 USA; 6 Department of Medicine, Emory University School of Medicine, Atlanta, GA 30307 USA; 7 The Wallace H. Coulter Department of Biomedical Engineering, Georgia Institute of Technology, Atlanta, GA 30332 USA; 8 Mathematical Sciences Department, Worcester Polytechnic Institute, Worcester, MA 01609 USA

**Keywords:** Vulnerable plaque, OCT, IVUS, Morphological index, Patient-specific model, FSI

## Abstract

**Background:**

Coronary plaque vulnerability prediction is difficult because plaque vulnerability is non-trivial to quantify, clinically available medical image modality is not enough to quantify thin cap thickness, prediction methods with high accuracies still need to be developed, and gold-standard data to validate vulnerability prediction are often not available. Patient follow-up intravascular ultrasound (IVUS), optical coherence tomography (OCT) and angiography data were acquired to construct 3D fluid–structure interaction (FSI) coronary models and four machine-learning methods were compared to identify optimal method to predict future plaque vulnerability.

**Methods:**

Baseline and 10-month follow-up in vivo IVUS and OCT coronary plaque data were acquired from two arteries of one patient using IRB approved protocols with informed consent obtained. IVUS and OCT-based FSI models were constructed to obtain plaque wall stress/strain and wall shear stress. Forty-five slices were selected as machine learning sample database for vulnerability prediction study. Thirteen key morphological factors from IVUS and OCT images and biomechanical factors from FSI model were extracted from 45 slices at baseline for analysis. Lipid percentage index (LPI), cap thickness index (CTI) and morphological plaque vulnerability index (MPVI) were quantified to measure plaque vulnerability. Four machine learning methods (least square support vector machine, discriminant analysis, random forest and ensemble learning) were employed to predict the changes of three indices using all combinations of 13 factors. A standard fivefold cross-validation procedure was used to evaluate prediction results.

**Results:**

For LPI change prediction using support vector machine, wall thickness was the optimal single-factor predictor with area under curve (AUC) 0.883 and the AUC of optimal combinational-factor predictor achieved 0.963. For CTI change prediction using discriminant analysis, minimum cap thickness was the optimal single-factor predictor with AUC 0.818 while optimal combinational-factor predictor achieved an AUC 0.836. Using random forest for predicting MPVI change, minimum cap thickness was the optimal single-factor predictor with AUC 0.785 and the AUC of optimal combinational-factor predictor achieved 0.847.

**Conclusion:**

This feasibility study demonstrated that machine learning methods could be used to accurately predict plaque vulnerability change based on morphological and biomechanical factors from multi-modality image-based FSI models. Large-scale studies are needed to verify our findings.

## Background

Plaque rupture is a main cause of arterial thrombosis which could lead to stroke or heart attack [1]. Early detection of rupture-prone plaques will be an important advance in atherosclerotic disease prevention. American Heart Association (AHA) published a series of reports on the definitions of different lesions of atherosclerosis [2, 3]. The AHA plaque classification scheme based on qualitative histology has been considered as the standard and guideline for plaque research for decades. Burke et al. and Arbustini et al. indicated that a fibrous cap thickness < 65 μm was an important threshold to identify vulnerable plaques [4, 5]. A more quantitative classification of atherosclerotic plaques was given based on a large number of histological data and analysis [6, 7]. Kolodgie et al. pointed out that plaque prone to rupture (also called thin-cap fibroatheroma) had three main characteristics: large lipid-rich necrotic core, higher prevalence of macrophage infiltration in fibrous cap, and a fibrous cap with thickness < 65 μm [8]. Naghavi et al. indicated that the quantitative characteristics of vulnerability could contribute to assessment of vulnerable plaques [9].

In addition to morphological characteristics, researchers have also been investigating plaque vulnerability from biomechanical point of view. Richardson et al. first studied the relationship between plaque wall stress (PWS = plaque maximum principal stress) and lesion morphology through biomechanical analysis [10]. Subsequently, 2D cross-sectional plaque model was employed to study the relationship between plaque rupture and stress, particularly the peak circumferential stress [11–14]. These studies established that a peak circumferential stress of 300 kPa was the threshold for plaque rupture, and this value has been widely used as the threshold stress value for plaque vulnerability. It was commonly believed that the peak circumferential stress in cap would exceed 300 kPa when the cap thickness was < 65 μm, which was confirmed by the results of 3D coronary model using ex vivo computed tomography (CT) images [15]. In order to obtain better quantification of plaque vulnerability, Tang et al. proposed the morphological plaque vulnerability index (MPVI) based on morphological characteristics of plaque, and found that MPVI was significantly correlated with mechanical factors [16]. Considering mechanical factors for vulnerability, many studies utilized wall shear stress (WSS) to predict plaque vulnerability behavior [17, 18]. Corban et al. constructed computational fluid dynamics (CFD) based on 20 patients and performed statistical analysis, showed that combining WSS and plaque burden at baseline could contribute to more accurate prediction of the change of plaque vulnerability from baseline to follow-up [19]. A study from Wang et al. calculated morphological and mechanical vulnerability indices of human coronary plaques using coronary fluid–structure interaction (FSI) models based on intravascular ultrasound (IVUS) images. Their correlation analysis using linear mixed-effects (LME) model suggested that both morphological and mechanical factors should be considered for better assessment of plaque vulnerability [20]. The idea that integrating mechanical and morphological factors in plaque vulnerability research has also been employed in the studies on predicting future plaque vulnerability. Another plaque vulnerability study using IVUS data from 40 patients at baseline and 12-month follow-up showed that PWS and WSS were largely independent of each other (*P* = 0.001) and interplay of PWS and WSS would govern the changes of plaque [21]. The LME model was also used in their study, and all statistical analyses were completed both in SPSS and Wang et al. used generalized linear mixed regression model (GLMM), support vector machine (SVM) and random forest (RF) method and stress/strain data computed from IVUS-based FSI models to predict changes of plaque vulnerable indices using [22]. Their results showed that combining morphological and mechanical factors could lead to higher prediction accuracy, but optimal predictors for different methods varied.

Although IVUS image is currently extensively used in coronary plaque research and clinical practice, its resolution (150–200 μm) limited its ability to detect vulnerable plaque with thin cap. Optical coherent tomography (OCT) has high resolution (~ 10 μm) and is able to detect thin fibrous cap of vulnerable plaque and quantify cap thickness [23]. We proposed a 3D-FSI modeling approach combining IVUS and OCT (called IVUS + OCT model) for more accurate morphological and mechanical quantifications [24]. By merging IVUS and OCT together (overlapping segmented IVUS and OCT contours), we can obtain whole vessel morphology from IVUS and superior resolution from OCT and provide better accuracy for fibrous cap quantifications. Models based on IVUS + OCT images could provide more accurate stress/strain calculations for better plaque vulnerability assessment. The IVUS + OCT model with accurate cap thickness quantification and mechanical prediction could be very helpful for plaque research advancement.

Lack of patient follow-up data with high resolution (< 65 µm) to quantify plaque cap thickness, well-accepted plaque vulnerability indices to monitor vulnerability changes, and methods to predict future vulnerability behaviors with high prediction accuracies remain as challenges for researchers in this field. In this paper, patient follow-up IVUS, OCT and angiography data were acquired from two coronary arteries of one patient (f; age: 80). The 3D-FSI model based on IVUS and OCT follow-up data was constructed to obtain accurate coronary atherosclerotic plaque morphological and plaque stress/strain data, which in turn were used to investigate their relationships with plaque vulnerability. Thirteen key morphological factors from IVUS and OCT images and biomechanical factors from FSI models were extracted from 45 slices (with lipid cool and cap) at baseline for statistical analysis. Three morphological indices including lipid percentage index (LPI), cap thickness index (CTI) and morphological plaque vulnerability index (MPVI) based on lipid size and cap thickness were calculated and used as the quantitative measures for plaque vulnerability. The changes from baseline to follow-up of the three indices were treated as plaque vulnerability changes. Four machine learning methods: random forest (RF), discriminant analysis (DA), least square support vector machine (SVM) and ensemble learning (EL) were tested using all combinations of morphological and mechanical factors to predict the changes of plaque vulnerability indices. Prediction accuracies and specificities from the four methods were compared to identify optimal predictors and prediction methods for plaque vulnerability prediction. While this paper is only a pilot study, optimal predictors and prediction methods are the long-term goals of researchers and clinicians in the vulnerable plaque study field.

## Results

Among 13 single-factor predictors, wall thickness had the optimal prediction for LPI change (ΔLPI), minimum cap thickness was the optimal single-factor predictor for CTI change (ΔCTI) and MPVI change (ΔMPVI). Comparing prediction results of all combinations of 13 risk factors, it was found that combinational-factor predictor combining mechanical and morphological risk factors provided better predictions of ΔLPI, ΔCTI and ΔMPVI for all four prediction methods.

### Prediction of morphological indices using single-factor predictor

Using ΔLPI, ΔCTI and ΔMPVI as plaque vulnerability change, respectively, 13 key risk factors at baseline were used as predictors to feed four machine learning methods. Optimal single-factor predictors with the highest area under curve (AUC) for three morphological indices are listed in Table 1.

**Table 1:** The optimal single-factor predictors with AUC, sensitivity and specificity for three morphological indices using four machine learning methods

Index	ΔLPI		ΔCTI		ΔMPVI	
Method	Factor	AUC (Spe, Sen)	Factor	AUC (Spe, Sen)	Factor	AUC (Spe, Sen)
RF	Critical PWSn	0.856 (0.928, 0.749)	MinCT	0.749 (0.858, 0.555)	MinCT	**0.785 **(0.863, 0.656)
DA	Plaque area	0.875 (0.872, 0.677)	MinCT	**0.818 **(0.868, 0.613)	MinCT	0.752 (0.830, 0.646)
SVM	Wall thickness	**0.883 **(0.947, 0.653)	MinCT	0.697 (0.853, 0.334)	MinCT	0.727 (0.866, 0.404)
EL	Plaque area	0.776 (0.927, 0.767)	MinCT	0.719 (0.852, 0.530)	MinCT	0.766 (0.864, 0.654)

Bold indicates that AUC value is the largest in this columns

*Sen* sensitivity, *Spe* specificity

**Fig. 1:**
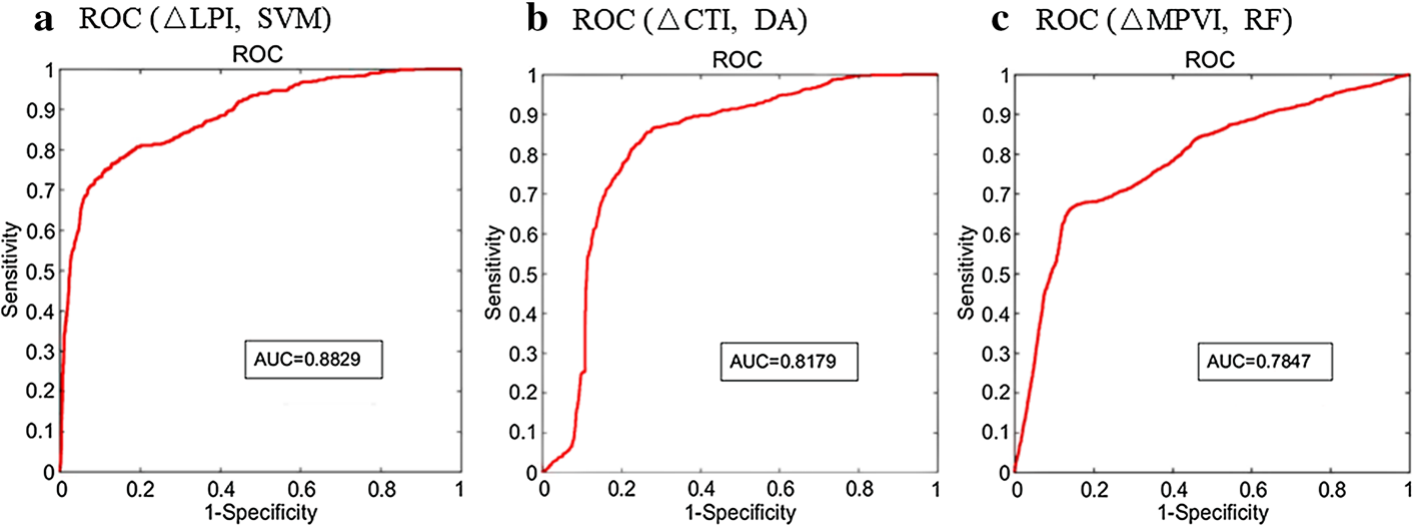
ROC obtained by optimal single-factor predictors and machine learning method with best performance for prediction of three morphological indices. **a** ROC of wall thickness predictor using SVM for ΔLPI prediction. **b** ROC of MinCT predictor using DA for ΔLPI prediction. **c** ROC of MinCT predictor using DA for ΔMPVI prediction

For ΔLPI prediction, baseline critical plaque wall strain (critical PWSn), plaque area, wall thickness and plaque area were the optimal single-factor predictor for RF, DA, SVM and EL with AUC 0.856, 0.875, 0.883 and 0.776, respectively. Although SVM had the best AUC, RF had the highest sum of sensitivity and specificity (1.677). In addition to that, its AUC is close to that of SVM (< 5%). For ΔCTI prediction, minimum cap thickness (MinCT) was the optimal single-factor predictor for all four machine learning methods (see Fig. 1). DA had the best AUC (0.818) and highest sum of sensitivity and specificity (1.481). For ΔMPVI prediction, MinCT was also the optimal predictor for all machine learning methods. RF had the best AUC (0.785) and highest sum of sensitivity and specificity (1.519). Definitions and calculation formulas for prediction accuracy and specificity are given in the Methods section. For each machine learning method, the AUC of ΔLPI were better than ΔCTI and ΔMPVI.

### Prediction of morphological indices using combinations of risk factors

All combinations of baseline 13 risk factors were used as predictors for three vulnerability indices using four machine learning methods. The optimal combination of risk factors with the highest AUC was identified. The optimal predictors of four machine learning methods for three vulnerability indices are shown in Table 2.

**Table 2:** The optimal combinational-factor predictors with AUC, sensitivity and specificity for three morphological indices using four machine learning methods

Index	ΔLPI		ΔCTI		ΔMPVI	
Method	Predictor	AUC (Spe, Sen)	Predictor	AUC (Spe, Sen)	Predictor	AUC (Spe, Sen)
RF	LP MeanCT Critical WSS Cap PWS	0.931 (0.971, 0.642)	MinCT Critical PWSn Critical WSS Cap WSS	0.826 (0.923, 0.555)	MinCT Plaque area Critical PWS	**0.847 (0.855, 0.583)**
DA	LP Wall Thickness Critical PWS Cap PWSn	0.957 (0.935, 0.920)	MinCT MeanCT Critical PWS Cap PWS Cap PWSn	**0.836 (0.823, 0.676)**	MinCT MeanCT Critical PWSn Cap PWSn Cap PWS	0.812 (0.831, 0.511)
SVM	LP Critical WSS Cap PWS Cap PWSn Cap WSS	**0.963 (0.974, 0.777)**	MinCT Lumen area Plaque area Critical PWSn	0.731 (0.926, 0.320)	MeanCT MinCT Plaque area Critical PWS Critical PWSn	0.773 (0.862, 0.436)
EL	LP MeanCT Lumen area Critical WSS Cap PWSn	0.861 (0.972, 0.607)	MeanCT MinCT Cap WSS	0.781 (0.915, 0.464)	MinCT Plaque area Critical PWSn	0.794 (0.870,0.508)

Bold indicates that AUC value is the largest in this columns

*Sen* sensitivity, *Spe* specificity

**Fig. 2:**
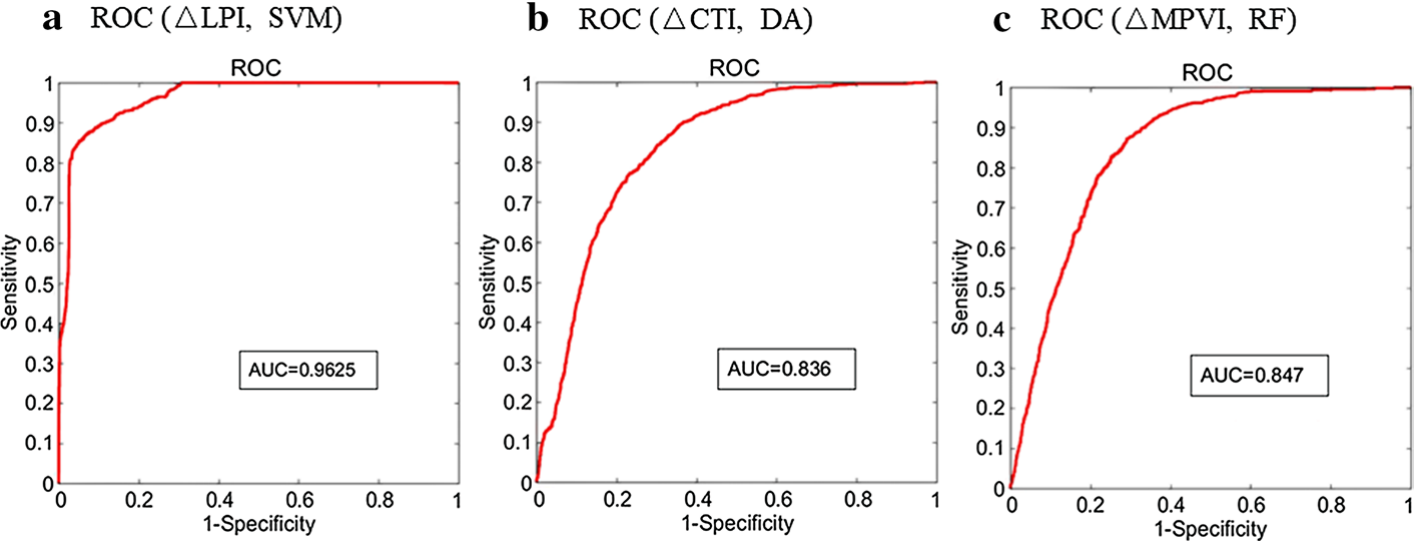
ROC obtained by optimal combinational-factor predictors and machine learning method with best performance for prediction of three morphological indices. **a** ROC of combinational-factor predictor (LP, critical WSS, cap PWS, cap PWSn and cap WSS) using SVM for ΔLPI prediction. **b** ROC of combinational-factor predictor (MinCT, MeanCT, critical PWS, cap PWS and cap PWSn) using DA for ΔLPI prediction. **c** ROC of combinational-factor predictor (MinCT, plaque area and critical PWS) using DA for ΔMPVI prediction

For ΔLPI prediction, the combination of lipid percentage (LP), critical WSS, PWS in fibrous cap (cap PWS), plaque wall strain in fibrous cap (cap PWSn) and WSS in fibrous cap (cap WSS) using SVM gave the best AUC (0.963). DA with the combination of LP, wall thickness, critical PWS and cap PWSn had the highest sum of sensitivity and specificity (1.855) within an AUC close to the optimal (< 5%). For ΔCTI prediction, the combination of MinCT, mean cap thickness (MeanCT), critical PWS, cap PWS and cap PWSn using DA gave the best AUC (0.836) and highest sum of sensitivity and specificity (1.499). For ΔMPVI prediction, the combination of MinCT, plaque area and critical PWS using RF achieved optimal AUC (0.847) and highest sum of sensitivity and specificity (1.438) (see Fig. 2). For each machine learning method, the AUC of ΔLPI were better than ΔCTI and ΔMPVI.

According to Tables 1 and 2, SVM has the optimal performance for ΔLPI prediction among the four machine learning methods. DA and RF show optimal prediction results for predicting ΔCTI and ΔMPVI among the four methods, respectively.

### Prediction difference between four machine learning methods

For vulnerability index change prediction using optimal single-factor predictor (see Table 1), the four machine learning methods could be ranked by AUC as follows: ΔLPI prediction: SVM > DA > RF > EL; ΔCTI prediction: DA > RF > EL > SVM; for ΔMPVI prediction: RF > EL > DA > SVM.

For ΔLPI and ΔCTI predictions, the AUC rankings of four methods are the same in both optimal combinational-factor predictors and optimal single-factor predictors, respectively. For ΔMPVI prediction with optimal combinational-factor predictors, the ranking of four methods using AUC is RF > DA > EL > SVM.

## Discussion

### Significance of high-resolution OCT image and multi-modality image-based models

The changes of coronary plaque wall thickness and fibrous cap thickness between baseline and follow-up were normally under 200 μm. The well-known cap thickness threshold value (65 μm) for vulnerable plaques is usually treated as an important standard for classification of morphological indices [7]. IVUS image resolution (150–200 μm) is not sufficient to measure cap thickness and plaque vulnerability changes accurately. OCT with its high resolution and IVUS with strong penetration could complement each other to give more accurate plaque morphology assessment, especially for cap thickness and lipid area. These improvements in turn could provide better stress/strain calculations [24]. Table 1 shows that most of the optimal single-factor predictor for three indices and four machine learning were associated with cap thickness. For ΔCTI and ΔMPVI, MinCT is the most important single-factor predictor. Even though ΔLPI is defined using lipid area, the optimal single-factor predictor is critical PWSn which is closely linked to cap thickness. Cap thickness and cap stress/strain are significant predictors for plaque vulnerability. IVUS + OCT-based FSI models led to more accurate vulnerability predictions.

### Errors in co-registration of IVUS and OCT data

The IVUS + OCT slices used for model construction were made by using lumen contours and plaque components from OCT and the vessel out-boundary from IVUS. Molony et al. used a dynamic programming algorithm to co-register IVUS and OCT data and reported that the co-registration to be accurate within 18º circumferentially and 0.64 mm longitudinally [24].

### Combination of morphological and mechanical factors could lead to more accurate predictions for three indices

A large number of vulnerable plaque studies concentrated on plaque morphology and hemodynamic factors, which provided better understanding and evaluation for the relationship between local fluid mechanics and plaque vulnerability. Corban et al. used IVUS-based CFD models and found that plaque locations with plaque burden > 40% and low WSS (defined as < 10 dynes/cm^2^) had significantly greater change in plaque area at follow-up [19]. In addition to hemodynamic risk factor WSS, PWS as structural risk factor was also used to predict plaque vulnerability. Costopoulos et al. extracted PWS from IVUS-based 2D structural mechanical models and WSS from CFD models to study changes of vulnerable plaque between baseline and 12-month follow-up [21]. Changes in plaque area, plaque burden, necrotic core (NC), fibrous tissue (FT), fibro-fatty tissue, and dense calcium were calculated for each co-registered frame [21]. By establishing a series of sophisticated FSI models, Tang et al. demonstrated that structural stress/strain, especially critical stress/strain may play an important role in plaque progression and vulnerability change [16, 28]. Despite the limitation of IVUS image resolution, some vulnerable plaque studies based on IVUS demonstrated that baseline mechanical risk factors improved prediction accuracy of vulnerability index change [20, 22]. Wang et al. constructed IVUS-based 3D-FSI coronary plaque models to obtain PWS, PWSn and WSS for correlation analysis and vulnerability prediction. The results indicated that critical PWS correlated with MinCT, CTI, MPVI with *r* = − 0.6414, 0.7852, and 0.7411, respectively (*p* < 0.0001) [20]. The combination of wall thickness, LA, plaque area, critical PWS, and MPVI was the best predictor using RF with the highest prediction accuracy 91.47% [22]. The FSI model with follow-up IVUS and OCT data could provide more accurate morphology and precise structure/fluid mechanics calculations [24, 25], which would improve the quantification of the plaque morphology change from baseline to follow-up and the prediction of plaque morphological indices. Because acquisition of baseline and follow-up IVUS + OCT data is difficult and the construction of 3D IVUS + OCT image-based FSI model is complicated and time-consuming, prediction study based on IVUS + OCT is rare in the existing literature. By comparing Tables 1 and 2, we could find that best combination of morphological and mechanical risk factors provided higher AUC and higher sum of sensitivity and specificity than optimal single-factor predictors. The AUC of optimal combinational-factor predictor is 8.5% higher than that of optimal single-factor predictor using ΔLPI and EI method. Using ΔLPI, the AUC of optimal combinational-factor predictor using SVM method achieved up to 96.3%. Combining morphological factors, fluid dynamics factors and structural mechanical factors demonstrated great ability in morphological indices prediction.

### Vulnerability indices and prediction methods

Three indices showed different morphological insights of plaque vulnerability changes. The changes of lipid area and cap thickness from baseline to follow-up were not completely consistent. Hence, the imbalances slice classes (‘index increase’ class vs. ‘index no increase’ class) for the three indices were not the same. There are differences in generalization ability, class imbalance, training speed between different machine learning methods. According to prediction results given in Tables 1 and 2, SVM was the best machine learning method for the prediction of ΔLPI; DA was the best method for the prediction of ΔCTI while RF was the best for ΔMPVI. For prediction using single-factor predictors, the maximum absolute error of prediction results achieved 12% between four machine learning methods for each index. For prediction using combinational-factor predictors, the maximum absolute error of prediction results achieved 10% between four machine learning methods for each index. The training speed of EL is the lowest among four machine learning methods because of serial processing of EL with boosting algorithm.

### Limitations

(a) Sample size: the sample size was small in our studies since it is challenging to obtain follow-up image data including IVUS, OCT and angiography. Only two arteries from one patient were used to make the follow-up FSI models. To compensate for this limitation, IVUS + OCT slice rather than artery was used as the prediction unit in our analysis to demonstrate the procedure of multi-modality image-based prediction analysis, and preliminary results were presented. (b) Neither IVUS nor OCT is ECG-gated, so it is likely that co-registered images were acquired at different time points in the cardiac cycle. That is a common problem in extensive OCT imaging and modeling. (c) Modeling limitation: many modeling conditions and assumptions could affect model stress/strain calculations, such as pressure conditions, patient-specific material properties, residual stress, cardiac motion and others. Our modeling procedure will be improved when data become available. Some parts of the complicated process in this study were performed manually including image co-registration, contour extraction, modeling procedure, etc., which were very time-consuming. (d) Prediction methods: only four machine learning methods were utilized in this study. In fact, different methods could affect the prediction analysis in many ways, such as prediction accuracy, reliance of imbalanced sample, operating time, loading space, etc. We can search for more appropriate methods including deep learning methods when large sample size of data could be obtained. This is a feasibility study to show that combining multi-modality image-based FSI model and machine learning method could potentially predict changes of vulnerability index more accurately. Large-scale patient studies are needed for further validation.

## Conclusion

IVUS + OCT data provided accurate cap thickness and better plaque morphology which led to better stress/strain calculations using IVUS + OCT-based FSI models and more accurate vulnerability prediction using machine learning predictive methods. Combination of 13 morphological and mechanical factors could lead to higher accuracy for vulnerability change predictions.

## Methods

### IVUS and OCT data acquisition and processing

**Fig. 3:**
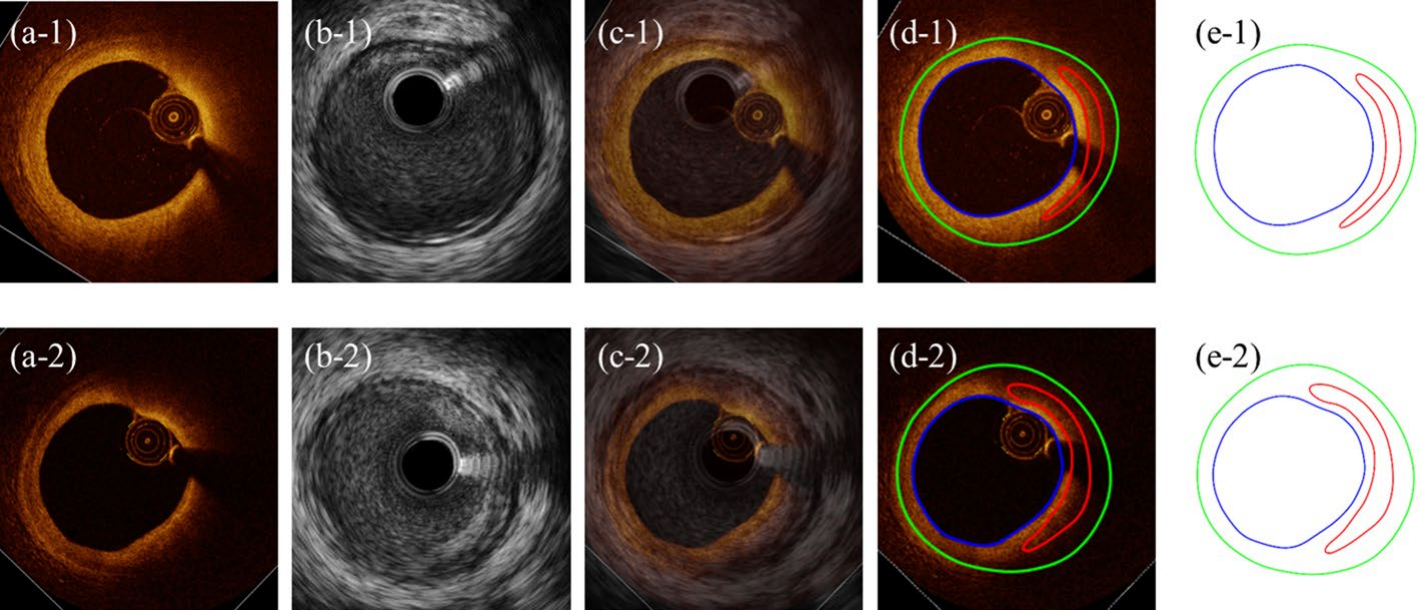
One sample of paired IVUS and OCT images at baseline and follow-up with segmented contours. Upper row: IVUS and OCT images were obtained at baseline; Lower row: IVUS and OCT images were obtained at follow-up. All images are from the same location of RCA. From left to right: (**a**-*) OCT image, (**b**-*) IVUS image, (**c**-*) IVUS image is overlaid with the paired OCT image, (**d**-*) OCT image with segmented contours, (**e**-*) Segmented IVUS + OCT contours. The symbol asterisk represents 1 and 2. Blue: lumen contour; green: out-boundary; red: lipid contour

This is a prospective study. Baseline and 10-month follow-up in vivo IVUS/OCT/angiography data were acquired from left circumflex coronary artery and right coronary artery (RCA) of one participant (female; age: 80) at Cardiovascular Research Foundation (CRF) using protocol approved by the local institute and informed consents were obtained from the patient. This patient was selected for our biomechanical and machine learning methodology preliminary study from a CRF data set where patients were with stable angina pectoris undergoing percutaneous coronary intervention (PCI). Patients with acute coronary syndrome, severe calcified lesion, chronic total occlusion or chronic kidney disease (Cr > 1.5 mg/dl) were excluded. The IVUS/OCT/angiography data were acquired at baseline and follow-up following the same procedures. IVUS catheter (Boston Scientific/SCIMED Corporation) with an automatic pullback speed of 0.5 mm/s was performed to acquire IVUS images. Then, OCT catheter (St. Jude, Minnesota, MN, USA) was also traversed to same region of interest and an automatic pullback at 20 mm/s was performed. The catheters’ positions were tracked by angiography and aortic pressure were recorded with pressure sensor in catheter. As IVUS and OCT images were not recorded using the same catheter in one pullback, they must be co-registered. That is, the IVUS and OCT images acquired from the same plaque locations were paired. Vessel branches were used as the first landmarks, and features common and visible in both IVUS and OCT (lumen area and eccentricity, lumen narrowing, lipid core, catheter position and calcifications) were used as the second landmarks for matching IVUS and OCT slices. At the same segment between landmarks, the frequency ratio of image generation between OCT and IVUS was ~ 12. Furthermore, the merged IVUS + OCT data at 2 time points (baseline and follow-up) also were registered for plaque progression measurements between baseline and follow-up. Co-registration was performed manually and independently by three experts based on relevant branches and landmarks described above [24]. The average was taken when the results from experts were inconsistent. After longitudinal and circumferential registration, a total of 105 paired IVUS and OCT images denoted as IVUS + OCT data were obtained at both baseline and follow-up. Forty-five slices with lipid core and fibrous cap were selected as machine learning sample database for vulnerability prediction study. IVUS + OCT data at baseline and follow-up were further matched one-by-one to quantify the change of plaque morphology. Three plaque compositions were considered in segmentation for IVUS + OCT data: lipid-rich necrotic core (short for lipid), calcification and other vessel tissue (fibrotic, fibro-fatty, etc.). Segmentation was performed by ImageJ 1.52v software. Small-size plaque components were neglected for simplification. Figure 3 gives samples of paired IVUS and OCT images at baseline and follow-up and corresponding segmented IVUS + OCT contours. Paired IVUS and OCT contours were merged together to make IVUS + OCT slices with IVUS providing out-boundary contours, and OCT providing lumen and plaque component contours.

### 3D coronary plaque geometry reconstruction

**Fig. 4:**
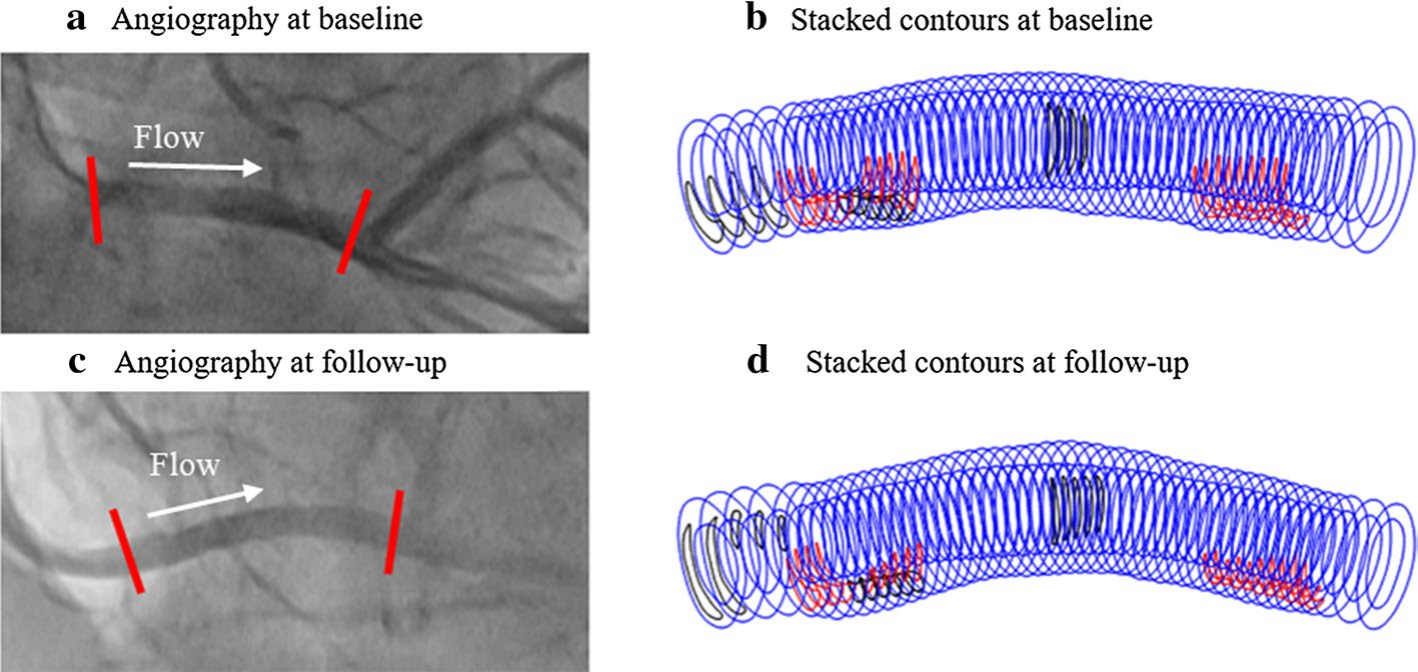
Angiography images and stacked contours of 3D RCA segment with minimum centerline curvature at baseline and follow-up. **a** The angiography image with minimum pressure at baseline. **b** Stacked contours plot with minimum centerline curvature at baseline. **c** The angiography image with minimum pressure at follow-up. **d** Stacked contours plot with minimum centerline curvature at follow-up. Contour color: red, lipid; black, calcification; blue, lumen and out-boundary

3D coronary plaque geometries were reconstructed by integrating the segmented IVUS + OCT contours and the corresponding angiography images. The 3D centerline of coronary geometry was extracted from angiography images and IVUS + OCT slices were stacked on it perpendicularly (see Fig. 4). Figure 4 shows the angiography images and stacked contours of 3D vessel segment with minimum centerline curvature at baseline and follow-up. Coronary arteries have cyclic bending caused by cardiac contraction/expansion. Hence, coronary movement extracted from angiography movie was applied in the FSI model to recover its cyclic movement. Aortic pressure measurements (max, min pressure: 136, 88 mmHg) were obtained at aortic ostium by the pressure sensor. Pulsating pressure conditions were prescribed at the inlet and outlet of the vessel segment (see Eq. (4) below). Axial shrinkage was set at 5% in our models since atherosclerotic vessels were stiffer than healthy vessels. More details of our model reconstruction are provided in our previous papers [24, 25].

### The complete 3D-FSI model

For our FSI model, blood was assumed to be Newtonian and incompressible. The Navier–Stokes equations with arbitrary Lagrangian–Eulerian (ALE) formulation were employed as the governing equations. No-slip conditions were assumed at the interface between fluid and structure. Natural traction equilibrium conditions were assumed at all interfaces. The governing equations and boundary conditions for the FSI model are as follows:

1 $$\rho \left(\partial \mathbf{u}/\partial t+\left(\left(\mathbf{u}-{\mathbf{u}}_{g}\right)\cdot \nabla \right)\mathbf{u}\right)=-\nabla p+\mu {\nabla }^{2}\mathbf{u},$$

2 $$\nabla \cdot \mathbf{u}=0,$$

3 $${\mathbf{u}|}_{\Gamma }=\partial \mathbf{v}/\partial t, { \partial \mathbf{u}/\partial n|}_{\mathrm{inlet},\mathrm{outlet}}=0,$$

4 $${p|}_{\mathrm{inlet}}={p}_{\mathrm{in}}\left(t\right), { p|}_{\mathrm{outlet}}={p}_{\mathrm{out}}\left(t\right),$$

5 $$\rho {v}_{i,tt}={\sigma }_{ij,j}, i,j=\mathrm{1,2},3, \mathrm{sum\,over}\, j,$$

6 $${\varepsilon }_{ij}={(v}_{i,j}+{v}_{j,i}+{v}_{\alpha ,i}{v}_{\alpha ,j})/2,\,i,j=1, 2 ,3,$$

7 $${{\sigma }_{ij}^{r}\cdot {n}_{j}|}_{\mathrm{interface}}={{\sigma }_{ij}^{s}\cdot {n}_{j}|}_{\mathrm{interface}},$$

8 $${\mathbf{x}}_{{{\text{center}}}} = {\mathbf{x}}_{{{\text{bending}}}} \left( {\text{t}} \right),$$

where **u** is fluid velocity, $p$ is pressure, **u**_g_ is the mesh velocity, $\mu$ is the dynamic viscosity, $\rho$ is density, $t$ stands for time, $\Gamma$ stands for vessel inner boundary, f_*j_ stands for derivative of f_*_ with respect to the *j*th variable, ${\varvec{\sigma}}$ is the stress tensor (superscripts indicate different materials), ${\varvec{\varepsilon}}$ is the strain tensor, ***v*** is the solid displacement vector, superscript letters *r* and *s* were used to indicate different materials, **x**_center_ is the position of vessel center line, and **x**_bending_ is the imposed cyclic bending condition derived from patient angiography movie.

### Constitutive material models for vessel tissue and plaque components

Coronary vessel material (fibrous tissue) was assumed to be hyperelastic, anisotropic, nearly incompressible and homogeneous. Plaque components (lipid core and calcification) were assumed to be hyperelastic, isotropic, nearly incompressible and homogeneous. The Mooney–Rivlin material models were used to describe the mechanical properties of vessel, fibrous tissue and plaque components. The following formulas are the strain energy density functions for isotropic and anisotropic Mooney–Rivlin materials, respectively:

9 $$W_{{{\text{iso}}}} = c_{1} \left( {I_{1} {-}3} \right) \, + \, c_{2} \left( {I_{2} {-}3} \right) \, + \, D_{1} \left[ { \, \exp \left( {D_{2} \left( {I_{1} {-}3} \right)} \right) \, {-}1} \right] + K\left( {J - 1} \right),$$

10 $$W_{{{\text{aniso}}}} = W_{{{\text{iso}}}} + \, \left( {K_{1} /K_{2} } \right)\left\{ {\exp \left[ {K_{2} \left( {I_{4} - 1} \right)^{2} } \right] - 1} \right\},$$

where $$I_{1} = \sum {C_{ii} } ,I_{2} = {\raise0.7ex\hbox{$1$} \!\mathord{\left/ {\vphantom {1 2}}\right.\kern-\nulldelimiterspace} \!\lower0.7ex\hbox{$2$}}\left[ {I_{1}^{2} - C_{ij} C_{ij} } \right]$$ *I*_1_ and *I*_2_ are the first and second invariants of right Cauchy–Green deformation tensor ***C*** = [*C*_*ij*_] = **X**^T^**X**, **X** = [X_ij_] = [∂x_i_/∂*a*_*j*_], (*x*_*i*_) is current position, (*a*_*i*_) is original position, *I*_4_ = *C*_*ij*_(**n**_c_)_*i*_(**n**_c_)_*j*_, **n**_*c*_ is the unit vector in the circumferential direction of the vessel, *J* is the Jacobian of the deformation gradient tensor, *K* is the Lagrange multiplier for the incompressibility, *c*_1_, *c*_2_, *D*_1_, *D*_2_, *K*_1_ and *K*_2_ are material parameters [24, 26]. Material constants of isotropic Mooney–Rivlin model from existing literature were used [24, 27]: lipid: *c*_1_ = 0.5 kPa, *c*_2_ = 0 kPa, *D*_1_ = 0.5 kPa, *D*_2_ = 1.5; calcification: *c*_1_ = 92 kPa, *c*_2_ = 0 kPa, *D*_1_ = 36 kPa and *D*_2_ = 2; vessel/fibrous tissue: *c*_1_ = − 278.7 kPa, *c*_2_ = 24.35 kPa, *D*_1_ = 133.7 kPa, *D*_2_ = 2, *K*_1_ = 7.19 kPa, *K*_2_ = 23.5 [27].

### 3D-FSI model solution method

**Fig. 5:**
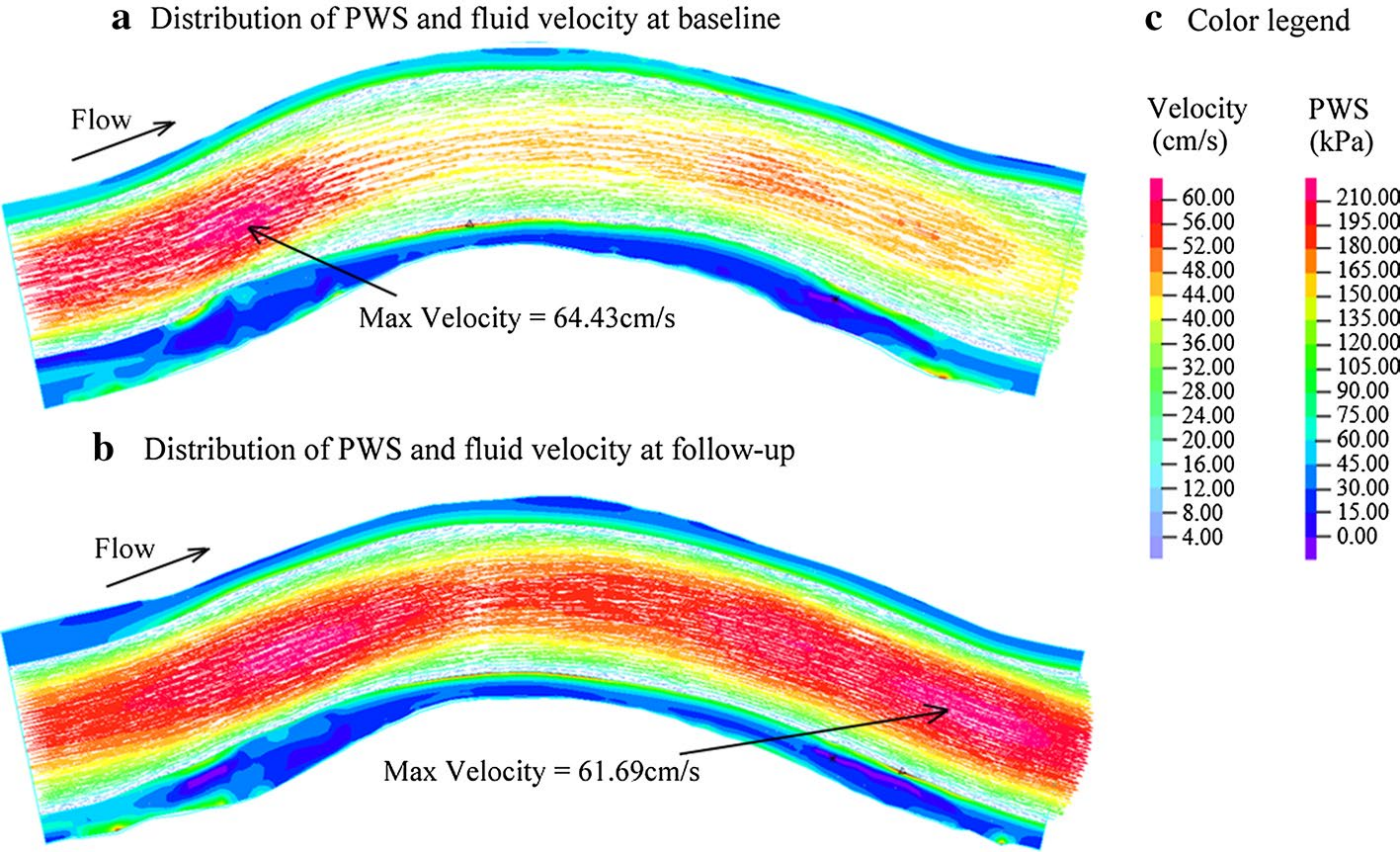
Distribution of PWS and fluid velocity from FSI model of RCA at baseline and follow-up. The mechanical state of vessel is displayed by band plot of PWS on longitudinal cross-section. Blood flow is shown by vector plot of fluid velocity on longitudinal cross-section. **a** Distribution of PWS and fluid velocity at baseline; **b** distribution of PWS and fluid velocity at follow-up; **c** color legend

The FSI models were solved by a finite element software ADINA 9.0 (Adina R&D, Watertown, MA, USA) following our established procedures [24]. ADINA uses unstructured finite-element methods for both fluid and solid models. Mesh analysis was performed by refining mesh density by 10% until changes of solutions became < 2%. Nonlinear incremental iterative procedures were used to solve FSI model. Three cardiac cycles were simulated for our FSI model and the solution in the third period was taken as the final result since the solutions for the second and third cycles became almost identical. Figure 5 shows distributions of PWS and fluid velocity in RCA under maximum pressure conditions at baseline and follow-up.

### Data extraction and plaque measurements

**Fig. 6:**
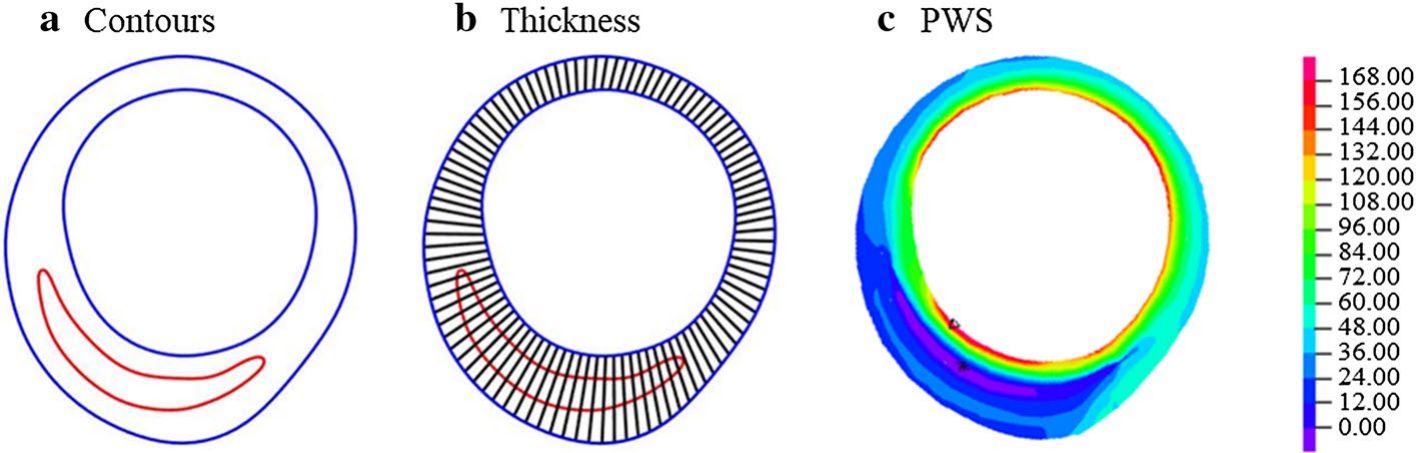
Morphological and mechanical factors are extracted from one sample slice. **a** Contours. Color: red, lipid; blue, lumen and out-boundary. **b** Thickness definition. Wall thickness and cap thickness were extracted from the connected lines. **c** PWS distribution on the circumferential cross-section paired with the slice (unit: kPa)

Out of 105 IVUS + OCT slices matched at baseline and follow-up, 45 slices containing lipid/fibrous cap were selected for subsequent prediction analysis. Morphological and mechanical factors of 45 matched slices (2 × 45 = 90 slices in total) were extracted from IVUS + OCT data and 3D-FSI models, respectively. Each slice contained 100 evenly spaced nodal points taken on the lumen. The lumen nodal point was connected to a corresponding point on vessel out-boundary (see Fig. 6). The length of the connecting line is defined as the wall thickness. If the line passes through a lipid or calcification region, the distance between lumen nodal point and the point that the line first time meets the lipid or calcification is defined cap thickness. The average and minimum values of cap thickness from one slice were obtained and recorded as mean cap thickness (MeanCT) and minimum cap thickness (MinCT), respectively.

The area bounded by the lipid contour in a slice was recorded as lipid area. The area enclosed by lumen contour was denoted as lumen area. The area between lumen and out-boundary was defined as plaque area. Lipid percentage (LP) and the plaque burden were defined by the following formulas:

11 $${\text{Lipid percentage }}\left( {{\text{LP}}} \right) = ({\text{lipid area/plaque area}}) \times {1}00\% ,$$

12 $${\text{Plaque burden }} = \, [({\text{plaque area}})/({\text{plaque area}} + {\text{lumen area}})] \times {1}00\% .$$

Therefore, seven morphological risk factors used as predictors in this study included wall thickness, MeanCT, MinCT, LP, lumen area, plaque area, plaque burden.

Considering plaque rupture closely associated with mechanical conditions on lumen, wall shear stress (WSS), plaque wall stress (PWS) and plaque wall strain (PWSn) values at 100 lm nodal points of all slices were extracted from 3D-FSI model. Many studies have shown the significance of maximum cap stress for plaque vulnerability and rupture [28–30]. In our study, the maximum WSS, PWS and PWSn in cap of each slice were called as critical WSS, critical PWS and critical PWSn, respectively. The mean WSS, PWS and PWSn in the fibrous cap of each slice were denoted as cap WSS, cap PWS and cap PWSn, respectively. Hence, six mechanical risk factors used as predictors in this study included cap WSS, cap PWS, cap PWSn, critical WSS, critical PWS and critical PWSn.

### Definition of morphological plaque vulnerability indices

Morphological characteristics including lipid and fibrous cap are commonly used to evaluate the plaque vulnerability according to previous histological analysis [7, 8, 31, 32]. Virmani et al. and Kolodgie et al. revealed the importance of LP in plaque stability [6–8]. It is also established that 65 μm is the key characteristic of plaque rupture, 200 μm is the threshold of the thickness of the thin fibrous cap, and 300 μm is a vague speculation [33, 34]. Therefore, LP and cap thickness were considered as measures for plaque vulnerability. Then the lipid percentage index (LPI) and cap thickness index (CTI) were assigned to each slice at both baseline and follow-up based on the values of LP and MinCT, respectively. The criteria for the indices assignment are provided in Table 3.

**Table 3:** The classifications of lipid percentage index (LPI) and cap thickness index (CTI) for vulnerable plaque

LPI	The range of lipid percentage	CTI	The range of Min cap thickness
0	LP = 0% (no lipid)	0	No lipid
1	0% < LP < 5%	1	MinCT > 0.3 mm
2	5% ≤ LP < 15%	2	0.20 mm < MinCT ≤ 0.3 mm
3	15% ≤ LP < 25%	3	0.065 mm < MinCT ≤ 0.2 mm
4	25% ≤ LP < 100%	4	MinCT ≤ 0.065 mm

Taking both LPI and CTI into our consideration, morphological plaque vulnerability index (MPVI) is defined as follows:

13 $${\text{MPVI }} = {\text{ min }}\left( {{\text{LPI}},{\text{ CTI}}} \right).$$

For 45 paired slices, the change of LPI, CTI and MPVI from baseline to follow-up were used to measure the change of plaque vulnerability:

14 $$\vartriangle {\text{LPI}} = \, \left( {{\text{LPI}}\;{\text{at follow-up}}} \right) \, - \, \left( {{\text{LPI}}\;{\text{at baseline}}} \right).$$

boundary conditions for the FSI model

15 $$\vartriangle {\text{CTI}} = \, \left( {{\text{CTI}}\;{\text{at follow-up}}} \right) \, - \, \left( {{\text{CTI}}\;{\text{at baseline}}} \right).$$

16 $$\vartriangle {\text{MPVI }} = \, \left( {\text{MPVI at follow-up}} \right) \, - \, \left( {\text{MPVI at baseline}} \right).$$

Each morphological index could be treated as the prediction target in turn. For the sake of simplification, plaque slices were classified into two classes according to the change of each morphological index. Use ΔLPI as an example, if ΔLPI > 0, that means vulnerability index increase, then this slice would be labeled 1 (‘Label 1’ Class). Conversely, ΔLPI ≤ 0, it would be labeled -1 (‘Label-1’ Class). Similar rule was applied to labeling all selected slices were labeled for ΔCTI and ΔMPVI.

### Prediction for the change of plaque vulnerability indices

Four different machine learning methods were employed for the prediction of each plaque vulnerability index including least squares support vector machine (SVM), ensemble learning (EL), discriminant analysis (DA) and random forest (RF). Least squares SVM used Gaussian radial basis function as the kernel function and steepest descent method for searching optimal parameters. SVM and RF were performed by LS-SVMlab toolbox and RF toolbox, respectively. The DA method used the Classification Discriminant object which encapsulates a discriminant analysis classifier. EL used the Adaptive Boosting algorithm and the number of ensemble learning cycles was set to 100. EL employed the fitensemble function of MATLAB2015a (MathWorks, Inc.) with AdaBoostM1 algorithm. MATLAB2015a were used to compile and run all programs of four machine learning methods.

Due to the small sample size (45 slices), synthetic minority oversampling technique (SMOTE) was employed to extend the sample size in the class with fewer samples [35]. Then, the new sample after oversampling was used to train and test the four machine learning methods for predicting each plaque vulnerability index. A standard fivefold cross-validation procedure was performed using all selected slices. This procedure was repeated 100 times to stabilize the prediction results. The input parameters used in prediction methods are listed in Table 4. For each machine learning method, all possible combinations of 13 morphological and mechanical factors at baseline were fit to the method as predictors to determine the prediction accuracies. The optimal combination with highest prediction accuracy was identified for each plaque vulnerability index using each method. Receiver operating characteristic (ROC) analysis was used to evaluate the prediction performance among different methods, and area under curve (AUC), sensitivity and specificity were calculated. Defining vulnerability index change > 0 to be positive and ≤ 0 to be negative, sensitivity of prediction of prediction event (the given vulnerability index increased) using a given predictor is defined as the proportion of the true positive (TP) outcomes that are predicted to be positive. Similarly, specificity of prediction is defined as the proportion of the true negative (TN) outcomes that are correctly predicted to be negative. The formulas used in our calculations are given below:

17 $$\mathrm{Sensitivity}\hspace{0.17em}=\hspace{0.17em}\mathrm{total\,TP\,outcomes}/\left(\mathrm{total\,TP\,outcomes}\hspace{0.17em}+\hspace{0.17em}\mathrm{total\,FN\,outcomes}\right),$$

18 $$\mathrm{Specificity}\hspace{0.17em}=\hspace{0.17em}\mathrm{total\,TN\,outcomes }/ (\mathrm{total\,TN\,outcomes}\hspace{0.17em}+\hspace{0.17em}\mathrm{total\,FP\,outcomes}),$$

where FN = false negative, FP = false positive, respectively.

**Table 4:** Summary of input parameters used in FSI models and prediction methods

Image	Resolution (μm)	Image size	Field of view (mm^2^)	Pixel size (mm)
Image				
IVUS	150–200	512*512	9*9	0.01752
OCT	10–20	704*704	7.01*7.01	0.00996
Angiography	> 200	512*512	152*152	0.29688

Model parameters	*c*_1_ (kPa)	*c*_2_ (kPa)	*D*_1_ (kPa)	*D*_2_	*K* (kPa)	*K*_1_ (kPa)	*K*_2_
Material							
Tissue	− 278.7	24.35	133.7	2	13,157	7.19	23.5
Lipid	0.5	0	0.5	1.5	1250	–	–
Calcification	92	0	36	2	164,000	–	–
Pressures	Maximum = 136 mmHg, Minimum = 88 mmHg						

Prediction methods	Setting	Data processing	Validation
SVM	Kernel function: Gaussian radial basis function	Synthetic minority oversampling technique (SMOTE)	Fivefold cross-validation
RF	Number of tree: 20		
EL	Number of ensemble learning cycles: 100		
DA	Discriminant type: linear		

## Data Availability

The datasets used in the study are available from the corresponding author upon request.

## Abbreviations

AUC: Area under curve
CFD: Computational fluid dynamics
CTI: Cap thickness index
DA: Discriminant analysis
EL: Ensemble learning
FSI: Fluid–structure interaction
IVUS: Intravascular ultrasound
LP: Lipid percentage
LPI: Lipid percentage index
MeanCT: Mean cap thickness
MinCT: Minimum cap thickness
MPVI: Morphological plaque vulnerability index
OCT: Optical coherence tomography
PWS: Plaque wall stress
PWSn: Plaque wall strain
RCA: Right coronary artery
RF: Random forest
ROC: Receiver operating characteristic
SVM: Support vector machine
WSS: Wall shear stress
